# Organellar genome analysis of the marine red alga *Dasya binghamiae* (Dasyaceae, Rhodophyta) reveals an uncharacteristic florideophyte mitogenome structure

**DOI:** 10.1080/23802359.2016.1192515

**Published:** 2016-07-12

**Authors:** Diana A. Tamayo, Jeffery R. Hughey

**Affiliations:** Division of Mathematics, Science and Engineering, Hartnell College, Salinas, California, USA

**Keywords:** Mitogenome, mtDNA, plastid genome, red algae, systematics

## Abstract

Analysis of the marine red algal species *Dasya binghamiae* A.J.K. Millar using paired-end 36 bp Illumina sequences resulted in the assembly of its complete mitochondrial and plastid genomes. The mitogenome is 26,052 bp in length and contains 46 genes, and the plastome is 177,213 bp with 228 genes. The plastid genome shows high gene synteny with previously published Florideophyceae; however, the mitogenome contains several multigene rearrangements. These organellar data confirm the placement of *D. binghamiae* in *Dasya* C. Agardh.

The marine red algal family the Dasyaceae is distributed worldwide and consists of 14 genera and ∼150 species (Schneider & Wynne 2007; Guiry & Guiry 2016). A number of molecular phylogenetic studies addressing systematic questions in the Dasyaceae are published (de Jong et al. 1998; Choi et al. 2002; Yamagishi et al. 2014; Sjøtun et al. 2016), however there are no mitochondrial or plastid genomes reported for the family. Here we announce the organellar genomes of *Dasya binghamiae*, a Pacific North American species (Abbott & Hollenberg 1976) originally assigned to the monotypic genus *Pogonophorella* (Silva 1952), but later transferred to *Dasya* (Millar 1996).

DNA was extracted from *D. binghamiae* (UC2050572) collected from Tomales Bay, California (38°09'54.5”N, 122°54'56.6”W) using the protocol described by Lindstrom et al. (2011). The library was constructed and sequenced by the High-Throughput Genomics Center (Seattle, Washington, USA) yielding 18,729,858 filtered reads. The reads were assembled using denovo settings in CLC Cell 4.3.0 (^®^2015 CLC bio, a QIAGEN Company, Waltham, MA) and annotated following Hughey et al. (2014). Alignment of the *D. binghamiae* mitogenome to other Florideophyceae was accomplished with MAFFT (Katoh & Standley 2013). The maximum likelihood analysis was performed using RaxML (Stamatakis 2014) with default parameters in Galaxy (Giardine et al. 2005; Blankenberg et al. 2010; Goecks et al. 2010). The phylogenetic tree was generated with TreeDyn 198.3 at Phylogeny.fr (Dereeper et al. 2008).

The mitogenome of *D. binghamiae* (GenBank KX247283) is 25,052 bp in length, AT rich (77.4%), and contains 46 genes including 22 tRNA, 4 ribosomal proteins (rpl 16, rps 3, rps 11, rps 12), 2 rRNA (1 rnl, 1 rns), ymf39, and 17 genes involved in electron transport and oxidative phosphorylation. It contains the three conserved gene regions (ANS, CY, NR) found in Bangiophyceae and Florideophyceae (Yang et al. 2015), but differs in organization to published Florideophyceae by two inverted multigene rearrangements: 1) ribosomal proteins rps3 and rpl16 are situated with sdh2 and trnH; 2) rps12, SecY, and trnK are positioned near apocytochrome b. Phylogenetic analysis of the mitogenome of *D. binghamiae* places it in a well-supported clade with *Ceramium japonicum* (Figure 1).

**Figure 1.**
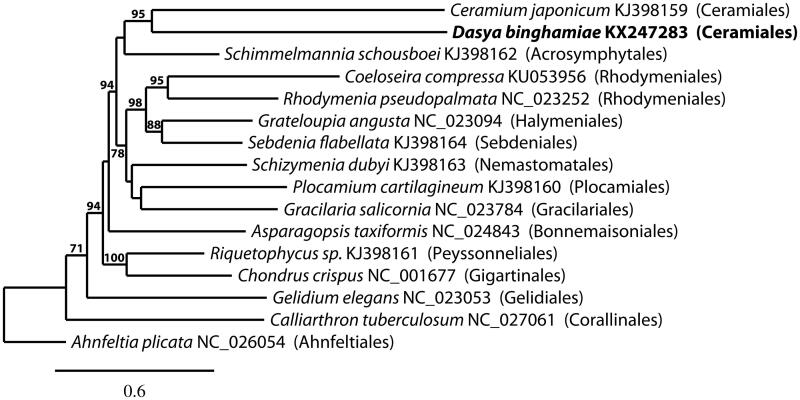
Maximum likelihood phylogram with *Dasya binghamiae* and other Florideophyceaen mitogenomes. Numbers along branches are RaxML bootstrap supports based on 1,000 nreps (<70% support not shown). The legend below represents the scale for nucleotide substitutions.

The complete plastid genome of *D. binghamiae* (GenBank KX247284) is 177,213 bp in length and contains 228 genes. The genome is AT rich (74.4%), and contains 19 small and 27 large ribosomal proteins, 29 photosystem I and II, 28 tRNA, 28 ycf, 13 orf, 10 phycobiliprotein, 8 cytochrome b/f complex, 8 ATP synthase, 3 rRNA, and 55 other genes. Plastid genome content and structure are similar to other Rhodymeniophycidae.
